# 2027: Racial differences in leukemia prognosis: New epidemiologic analysis

**DOI:** 10.1017/cts.2017.89

**Published:** 2018-05-10

**Authors:** Shuangge Ma, Yinjun Zhao, Yu Wang

**Affiliations:** Yale School of Medicine, New Haven, CT, USA

## Abstract

OBJECTIVES/SPECIFIC AIMS: Research on cancer difference is of significant scientific and practical value. For leukemia, the survival disadvantage of the Blacks has been suggested in multiple studies. However, the existing epidemiologic analysis has multiple technical limitations. The goal of this study is to more accurately quantify so as to better understand different sources of racial differences in leukemia survival. METHODS/STUDY POPULATION: A new statistical method, which is based on robust regression and resampling, is developed. Data are obtained from the SEER (Surveillance, Epidemiology, and End Results) database. Using the “classic” epidemiologic methods as well as the new method, analysis is conducted on the prognosis of 4 leukemia subtypes (ALL, CLL, AML, and CML) for 4 major racial groups (White, non-Hispanic White, Black, and Asian and Pacific Islander). RESULTS/ANTICIPATED RESULTS: After effectively removing differences caused by the observed clinicopathological and demographic factors, the survival disadvantage of the Blacks persists for the following patient groups: ALL and age>14, CLL and age>14, and ALL and age≤14. The quantitative results are significantly different from those from classic epidemiologic analysis. Such observed racial differences are more attributable to the unobserved risk factors and cancer disparity. DISCUSSION/SIGNIFICANCE OF IMPACT: This study provides a more effective and more direct quantification of racial difference in leukemia prognosis. The survival disadvantage of the Blacks which is observed for certain subtypes/age groups deserves further attention but should not be overstated. More data collection and analysis are needed to more accurately decipher racial differences in leukemia and other cancer types.

